# A TILLING by sequencing approach to identify induced mutations in sunflower genes

**DOI:** 10.1038/s41598-021-89237-w

**Published:** 2021-05-10

**Authors:** Valentina Fanelli, Kathie J. Ngo, Veronica L. Thompson, Brennan R. Silva, Helen Tsai, Wilma Sabetta, Cinzia Montemurro, Luca Comai, Stacey L. Harmer

**Affiliations:** 1 Department of Soil, Plant and Food Sciences (DiSSPA), University of Bari Aldo Moro, 70124 Bari, Italy; 2 Department of Plant Biology, University of California, Davis, CA 95616 USA; 3 National Research Council, Institute of Bioscience and BioResources-IBBR, 70124 Bari, Italy

**Keywords:** Agricultural genetics, Mutation, Computational biology and bioinformatics

## Abstract

The Targeting Induced Local Lesions in Genomes (TILLING) technology is a reverse genetic strategy broadly applicable to every kind of genome and represents an attractive tool for functional genomic and agronomic applications. It consists of chemical random mutagenesis followed by high-throughput screening of point mutations in targeted genomic regions. Although multiple methods for mutation discovery in amplicons have been described, next-generation sequencing (NGS) is the tool of choice for mutation detection because it quickly allows for the analysis of a large number of amplicons. The aim of the present work was to screen a previously generated sunflower TILLING population and identify alterations in genes involved in several important and complex physiological processes. Twenty-one candidate sunflower genes were chosen as targets for the screening. The TILLING by sequencing strategy allowed us to identify multiple mutations in selected genes and we subsequently validated 16 mutations in 11 different genes through Sanger sequencing. In addition to addressing challenges posed by outcrossing, our detection and validation of mutations in multiple regulatory loci highlights the importance of this sunflower population as a genetic resource.

## Introduction

Sunflower (*Helianthus annuus* L.) is a widely cultivated crop, with seed production estimated at about 51 million metric tons in the marketing years 2018/2019, making it the third largest oilseed crop in the world after soybean and rapeseed^1^. Most of its economic value comes from oil, which is predominantly used for alimentary purposes^2^. Cultivated sunflower is diploid (n = 17) and has a 3.6 Gb genome. The sunflower genome has undergone three episodes of polyploidization over the past 60 Myr and is dominated by repetitive elements. Staton et al.^3^ showed that about 77% of the sunflower genome is constituted of long terminal repeat retrotransposons (LTR-RTs) arising in a massive expansion that likely occurred in the past one million years. However, despite its large size, the whole sunflower genome was recently sequenced^4^.

The genome sequence will be a powerful tool for sunflower improvement via marker-assisted breeding and genomic selection. The generation of inbred lines is one of the principal aims of sunflower breeding programs and could be greatly accelerated by modern breeding techniques such as the production of doubled haploids. One method for the efficient generation of haploid plants is via manipulation of the centromere-specific histone H3 variant (CENH3). CENH3 replaces histone H3 in centromeric nucleosomes and recruits kinetochore proteins that bind microtubules to help coordinate chromosome segregation^5,6^. Hybridization of a plant with wild-type *CENH3* to one with a modified version of this protein can cause genome elimination and the spontaneous generation of a homozygous diploid via doubling of the haploid chromosome^7^. This type of doubled haploid induction can circumvent generations of inbreeding, greatly accelerating the breeding cycle^8^. Studies on *CENH3* genes have been already carried out in sunflower^9^.

In addition to its economic importance, sunflower provides an excellent model system for physiological, ecological, and evolutionary studies^10,11^. Sunflower plants are well known for their ability to track the sun, bending from east to west during the course of each day; in fact their name in many languages refers to this behavior. However, it is less recognized that they also bend from west to east during the night so that they face east before the sun rises^12^. We recently identified a role for the circadian clock in this process^13^. Circadian clocks are widespread in nature and generate self-sustained rhythms with an approximately 24-h period. In plants as in other eukaryotes, these rhythms are generated by cell autonomous oscillators that are comprised of multiple transcription factors acting in transcriptional feedback loops^14^. Clock genes not only control the expression of other clock genes but also regulate the expression of output genes, with at least one-third of the *Arabidopsis thaliana* transcriptome under circadian control^15^. Key components of the plant clock network include the LUX, ELF3, and ELF4 proteins, which together form the Evening Complex (EC) and repress expression of important day-phased genes^16^. The EC regulates many processes such as photoperiodic control of flowering time and the rhythmic control of hypocotyl growth^17,18^. LHY is another important transcription factor that regulates both circadian function and clock output pathways^14^.

Phototropic signaling pathways are also thought to play an important role in solar tracking movements^12^. Work mainly carried out in *Arabidopsis* has led to the model in which the activation of the phototropin photoreceptors by directional blue light leads to redistribution of the hormone auxin so that it accumulates on the shaded side of the stem and promotes bending towards light^19^. The directed transport of auxin throughout the plant is achieved by the asymmetric distribution of PIN-FORMED (PIN) auxin efflux carrier proteins^20^. There are eight *PIN* genes in *Arabidopsis*; *PIN3*, along with its homologs *PIN4* and *PIN7*, have been shown to be involved in both phototropic and gravitropic responses^21,22^.

Another trait both influenced by the circadian clock and of great agronomic importance is the timing of transition from vegetative to reproductive growth. Previous studies have implicated components of the flowering-time gene regulatory network in both the domestication and adaptation processes of wild sunflower to new environments. In particular, sunflower homologs of *FT* genes have been shown to be involved in these processes^23,24^. Sunflower has four *FT* paralogs, *HaFT1*, *HaFT2*, *HaFT3*, and *HaFT4*, but only *HaFT1*, *HaFT2,* and *HaFT4* are believed to be functional. Changes in other flowering time genes such as *HaBFT*, *HaTFL1* and *HaGA2OX2* may also have played important roles in sunflower domestication and adaptation^23,25^.

A molecular understanding of how these important physiological traits are controlled is very useful for the improvement of sunflower breeding. For example, insights into how the transition from vegetative to reproductive status is regulated and the ability to produce genotypes with different flowering times will be very important for targeted sunflower breeding programs. Until now, conventional breeding techniques such as interspecific hybridization have been used to improve sunflower^10^. Other methods such as classical mutagenesis have also been used to generate variability valuable in breeding programs; this is particularly useful if the trait of interest is not present in wild *Helianthus* species. For example, ethyl methanesulfonate (EMS) mutagenesis was used to obtain sunflower plants with different tocopherol profiles^26^ and enhanced phytoextraction ability^27^. A related approach is TILLING (Targeting Induced Local Lesions IN Genomes), a reverse genetic technique in which chemical mutagenesis is followed by screening for mutations using high-throughput methods. Two TILLING populations have previously been developed in sunflower^28,29^, and in both cases screening for mutations was performed by DNA digestion with mismatch-specific endonucleases followed by visualization on polyacrylamide gels. A more efficient method for identification of specific mutations in a mutagenized population is TILLING by sequencing, in which amplicons from mutagenized plants are pooled and then subjected to high-throughput sequencing. TILLING by sequencing provides important advantages, such as the ability to discover mutations present at a very low frequency in a sample (e.g. one mutant individual in a pool of 64 plants) and to identify the nature of any mutations at the time of discovery. This strategy has been successfully used in many organisms^30–38^.

In the current study, we aimed to identify sunflower plants with mutations in select genes involved in the circadian clock, auxin transport, control of flowering time, and chromosome segregation. We therefore used high throughput sequencing to identify gene alterations within a sunflower TILLING population (sunTILL) previously developed by Sabetta et al.^28^. Here we report on the isolation of target genes, our strategy for sequence generation and data analysis, the spectrum of identified mutations, and a phenotype in one of our identified mutants. Our results highlight the complexities of using this technique with a primarily outcrossing species and appropriate methods to deal with the consequent novel challenges.

## Results

### Candidate genes isolation

The genomic sequence of the sunflower *HaFT1*, *HaFT2*, *HaFT4*, *HaBFT*, *HaTFL1* and *HaGA2OX2* genes was known^23,25^, but no such information was available at the time for the other candidate genes in *H. annuus*, making their characterization necessary. We therefore used the sequences of *Arabidopsis thaliana* candidate proteins to identify several *H. annuus* genomic scaffolds encoding putative homologs. Each scaffold was analyzed in order to identify an ORF and to obtain a putative protein sequence. Only the scaffolds containing a complete ORF were kept for the following steps of analysis. Phylogenetic analysis was used to identify the likely orthologous genes and to differentiate members of the putative *HaPIN3, HaPIN4* and *HaPIN7* clade from the other *HaPIN* genes (Supplementary Fig. S1)*.* Thus, five putative *HaPIN3/4/7* and one *HaLHY* gene were identified. Moreover, a putative *HaELF3* was excluded since it did not cluster with the *ELF3* group (Supplementary Fig. S1). Expression of the homologous genes was verified by the presence of expressed sequence tags from the HaT13 and Ha412T4 transcriptomes (https://www.heliagene.org/). This in silico analysis led to the identification of four putative *HaLUX*, two putative *HaELF3*, six *HaELF4* and *ELF4-like*, five putative *HaPIN3/4/7*, two putative *HaCENH3*, and three putative *HaLHY* and *HaCCA1* genes in sunflower.

In order to confirm and, in some cases, to complete the sequence of the identified putative genes, their gDNAs and cDNAs were sequenced (Supplementary Data S1). All the gaps in the scaffolds were filled and in most cases the gene sequence was confirmed. In some cases, a few differences were also observed (Supplementary Fig. S2). cDNAs corresponding to the identified candidate genes were checked in samples collected at 3 different time points: ZT0 (dawn), ZT8 (mid-afternoon) and ZT16 (dusk). Expression of the putative *HaLUX*, *HaELF3*, and *HaELF4* genes was observed in the samples collected at ZT16, while the expression of the putative *HaLHY* gene was detected at ZT0 and ZT8. The expression of the putative *HaCENH3* genes was observed at all the time points, while the expression of *HaPIN3/4/7* was variable across the three time points.

**Figure 1:**
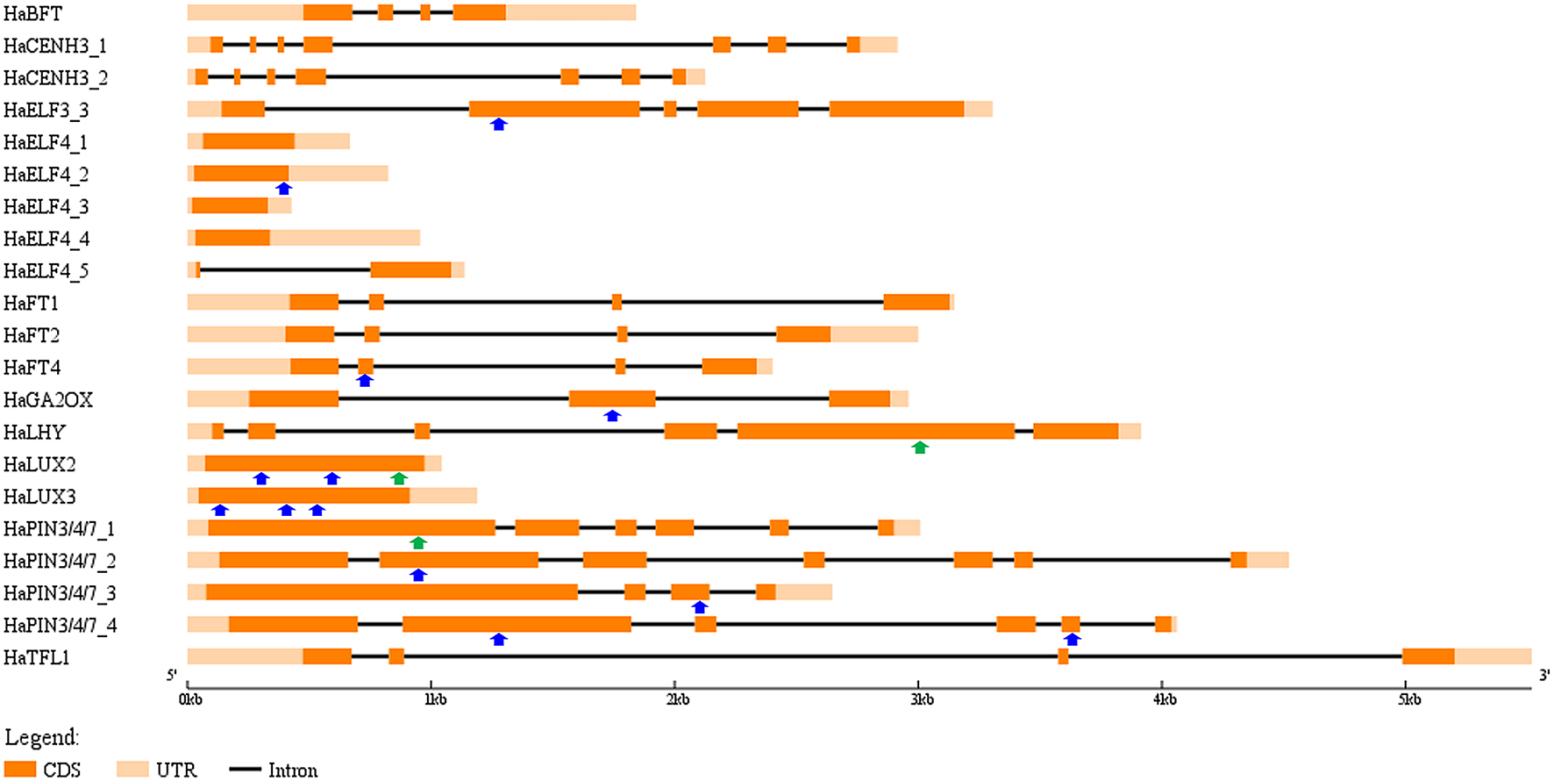
Structure of genes screened for mutations. Dark orange boxes indicate the exons. Lines linking exons represent introns. Light orange boxes indicate the UTRs. Blue and green arrows represent the position of validated heterozygous and homozygous mutations, respectively.

This analysis allowed us to screen the TILLING population for mutations in the following 21 sunflower genes: *HaFT1*, *HaFT2*, *HaFT4*, *HaBFT*, *HaTFL1*, *HaGA2OX*, *HaLUX2*, *HaLUX3*, *HaELF3_3*, *HaELF4_1*, *HaELF4_2*, *HaELF4_3*, *HaELF4_4*, *HaELF4_5*, *HaPIN3/4/7_1*, *HaPIN3/4/7_2*, *HaPIN3/4/7_3*, *HaPIN3/4/7_4*, *HaCENH3_1*, *HaCENH3_2*, and *HaLHY* (Fig. 1).

### Library preparation and sequencing

We subjected a previously developed sunflower TILLING population to the TILLING by sequencing strategy to detect single nucleotide polymorphisms in the above listed 21 genes. DNAs from 2048 M_2_ plants were pooled in 96 pools of 64 samples each through a tridimensional scheme whereby each sample was present in three different pools. Following the amplification of pools, ninety-six PCR pools were generated and used for the constitution of 96 Illumina libraries marked with 96 different barcoded adapters. The number of Illumina HiSeq lanes needed for sequencing was calculated from the total number of input bases divided by the minimum expected base sequence yield for a flow cell lane^30^. In order to obtain an estimated 100-fold coverage per amplicon, two lanes were sequenced on a HiSeq2500 Illumina system using the rapid run mode with the 100 bp paired-end reads method.

### Data analysis and mutation detection

**Figure 2:**
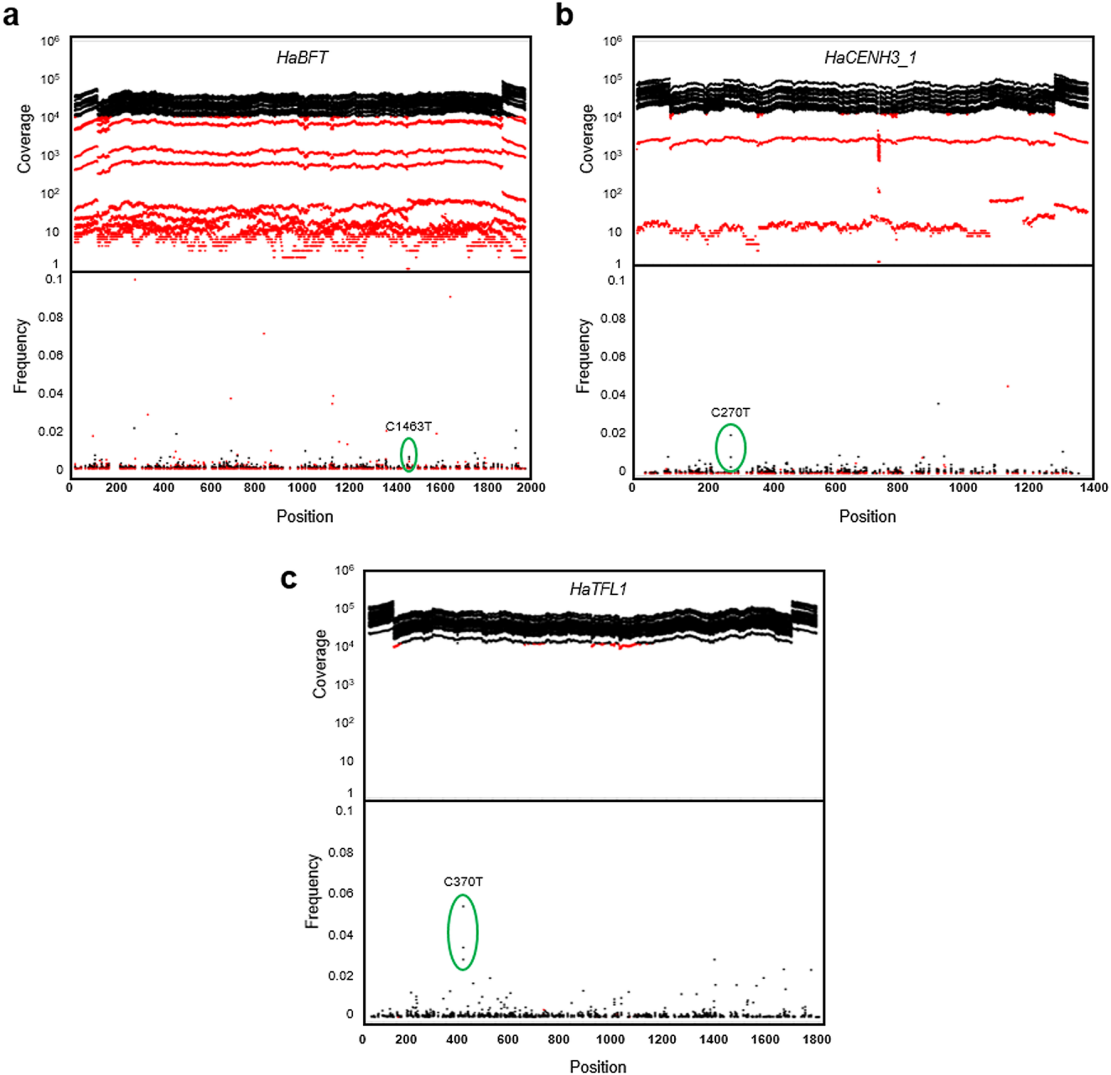
Effect of sequencing coverage on mutation detection and noise. Shown are results from 24 libraries constructed from PCR products generated from 512 three-dimensionally pooled genomic DNA samples. Sequence coverage and mutation frequency are shown for three genes: HaBFT **(a)**, HaCENH3_1 **(b)**, and HaTFL1 **(c)**. Each gene is represented in a separate panel that illustrates the per-base sequencing coverage in 24 libraries (top panels) and observed frequency of C > T changes (bottom panels). In both panels, base positions with coverage below 12,000-fold are represented in red to highlight the contribution of low-coverage bases to noise. Mutant individuals shared by three pools represented by three outliers are also indicated by green circles.

We obtained 203 and 205 million reads from the two lanes of sequence data. The average quality score per read (Phred score) was 38 for both lanes. About 70% of the reads passed the pre-processing (de-multiplexing and filtering) steps and were aligned to the target sequences. Coverage and nucleotide variation among genes and within targets were assessed using previously described methods^30^. Each amplicon showed high coverage at the gene termini, corresponding to the location of the primers used for PCR amplification, followed by a decline in coverage and then a gradual increase to average coverage levels. The coverage showed a high degree of variation between genes and pools (Fig. 2a–c), with the average sequencing depth of each amplicon ranging between 50- and 60,000-fold. Sequence variation is evident at virtually all positions; most of this is likely due to sequencing errors and represents noise. Alternatively, it could represent actual sequence changes, which could be natural polymorphisms or induced mutations. The screening of 21 genes from 2048 individuals yielded 3543 putative mutations, around 169 mutations in each gene.

In order to limit errors in mutation detection, an updated version of CAMBa, CAMBa2, was used to identify the candidate mutations^39,40^. CAMBa2 provides type and position of each candidate mutation, the predicted effect on gene function, and the F(t) score indicating the likelihood that the predicted mutation is real. Moreover, CAMBa2 assesses the severity of missense mutations based on BLOSUM62^41^ classifying them as not severe (NSM) or possible severe mutations (PSM). Of the 3,543 putative mutations, 68% were below the F(t) score of 2^30^. We filtered the mutation into three confidence classes according to F(t): a low confidence (likely noise) class with F(t) < 2 (n = 2420); a medium quality class with F(t) = 2–7.69 (n = 911), and high quality class with F(t) > 7.69 (n = 212). We first examined the aggregated medium and high quality dataset in which the screening of 21 genes from 2,048 individuals yielded 1123 putative mutations, an average of 53.5 per gene (Table 1). The number of detected mutations was correlated with tilled sequence length (Supplementary Fig. S3, Pearson r = 0.77, p = 1.4e−05). However, the correlation decreased when intron and exon space were considered individually (Supplementary Fig. S3). Particularly in the exon space, a considerable range in number of mutations between individual genes is evident even when comparing similarly-sized 2 kb regions (Supplementary Fig. S3).

**Table 1:** Summary of induced mutations with F(t) > 2 predicted by CAMBa2 in 21 genes from 2,048 individuals of the M_2_ sunflower population.

Gene	Canonical EMS mutations					Non-canonical EMS mutations				Tilled sequence	Tilled exon	Tilled intron	Total mutations	Total canonical mutations	Total non-canonical mutations	Total mutations intron	Total mutations exon
	Intron	Missense	Silent	Splice	Trunc	Intron	Missense	Silent	Splice								
HaBFT	1	2	9	0	1	12	23	32	2	1971	522	1449	82	13	69	13	69
HaCenH3_1_1	6	0	1	0	0	10	2	0	0	1479	222	1257	19	7	12	16	3
HaCenH3_1_2	5	3	0	0	0	26	2	14	0	1358	207	1151	50	8	42	31	19
HaCenH3_2	3	2	0	0	0	26	7	9	0	2025	351	1674	47	5	42	29	18
HaELF3_3	6	6	3	0	0	22	10	8	0	3303	1899	1404	55	15	40	28	27
HaELF4_1	0	1	10	0	0	0	1	7	0	2870	378	2492	19	11	8	0	19
HaELF4_2	0	3	6	0	0	0	6	5	0	1034	390	644	20	9	11	0	20
HaELF4_3	0	1	3	0	0	0	0	0	0	1295	312	983	4	4	0	0	4
HaELF4_4	0	1	2	0	0	0	5	13	0	1957	309	1648	21	3	18	0	21
HaELF4_5	7	3	1	0	1	15	7	6	0	1474	351	1123	40	12	28	22	18
HaFT1	7	3	2	0	0	43	5	2	0	3423	576	2847	62	12	50	50	12
HaFT2	11	3	2	1	0	57	6	18	1	3131	528	2603	99	17	82	68	31
HaFT4	10	2	2	0	0	7	6	4	0	2532	525	2007	31	14	17	17	14
HaGA2OX	7	3	5	0	0	9	12	13	0	3256	975	2281	49	15	34	16	33
HaLHY	8	16	6	0	0	23	47	34	1	4838	1926	2912	135	30	105	31	104
HaLUX2	0	5	2	0	1	0	3	5	0	1000	903	97	16	8	8	0	16
HaLUX3	0	4	1	0	0	0	5	7	0	1145	870	275	17	5	12	0	17
HaPIN3/4/7_1	4	7	7	0	0	4	13	20	0	2982	1830	1152	55	18	37	8	47
HaPIN3/4/7_2	8	7	6	0	1	41	34	7	0	5113	1830	3283	104	22	82	49	55
HaPIN3/4/7_3	2	2	6	0	0	4	3	7	0	3252	1851	1401	24	10	14	6	18
HaPIN3/4/7_4	21	16	11	0	1	45	28	26	0	4759	1860	2899	148	49	99	66	82
HaTFL1_1	1	0	0	0	0	6	6	5	0	1805	263	1542	18	1	17	7	11
HaTFL1_2	4	1	0	0	0	2	0	1	0	2093	259	1834	8	5	3	6	2

**Figure 3:**
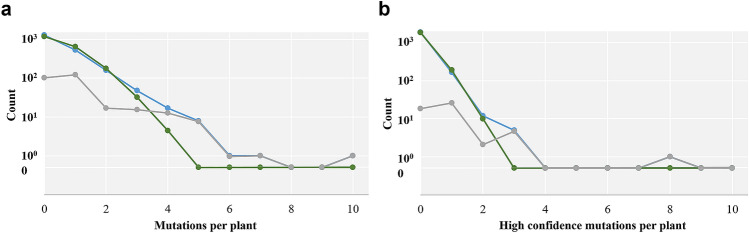
Comparison of Poisson modeling and observed polymorphism rates. The green line and points represent a typical outcome according to the Poisson distribution for 2048 individuals. In blue is the observed incidence. The grey line displays the absolute difference between modeled and observed. (**a)** Mutations with F(t) > 2. (**b)** Mutations with F(t) > 7.7.

EMS-induced mutations are typically more than 90% GC to AT transition. Only 295, about 26%, of alterations in our high- and medium-quality candidates fit this canonical expectation for EMS treatment-induced mutation. This is an unusually low ratio that does not differ from the 31% observed due to natural polymorphism^42^. In TILLING populations, induced mutations are rare and their occurrence can be modeled with the Poisson distribution. Individuals with multiple mutations are thus statistical outliers, noise-outliers, or genetic contaminants, either through seed or pollen. We examined the pattern of mutations in our population by comparing a typical Poisson outcome to the observed data (Fig. 3a,b, Supplementary Table S1). The TILLING population is enriched with individuals with multiple mutations (P of chi square = 0), indicating that around 130 mutations are present in individuals whose mutation load (> 3) at the sampled genes is difficult to explain by chance. The number of suspect mutations is likely higher because we observed many fewer individuals (526) with a single mutation than expected (649). The high confidence (F(t) > 7.69) set of 212 putative mutations had similar properties: it had 34% canonical EMS mutations and displayed a similar abnormal distribution of mutations among the individuals of the population (Fig. 3b, Supplementary Table S1. P of chi square = 0). These data suggest either systematic bias in our sequencing protocol or unexpected genetic variation in our TILLING population.

### Validation and mutation density

**Figure 4:**
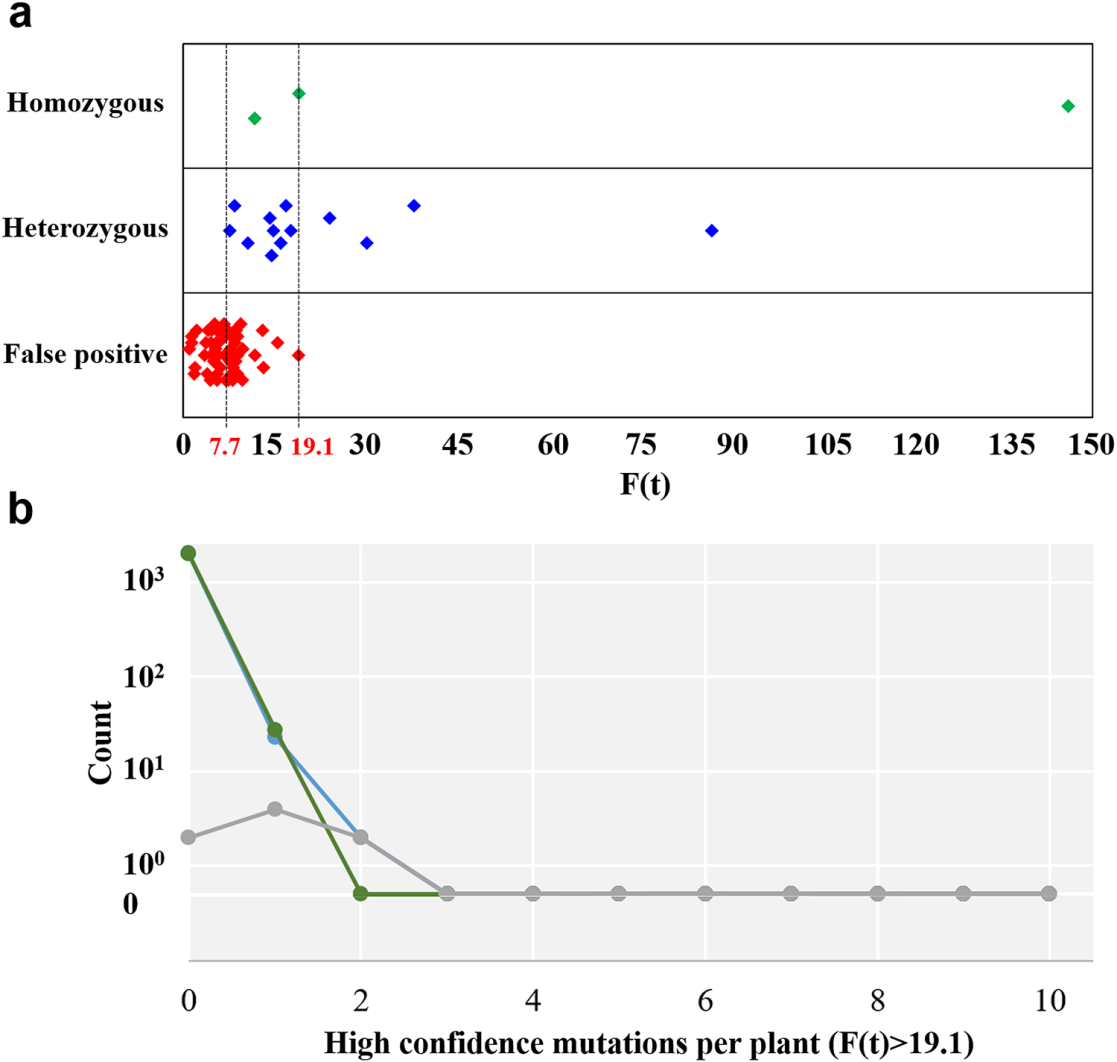
**(a)** F(t) score distribution of 69 mutations tested with Sanger sequencing. True positives (homozygous or heterozygous mutations) and false positives are indicated. The dashed lines indicate the thresholds of F(t) = 7.7 and F(t) = 19.1. (**b)** Comparison of Poisson modeling and observed polymorphism rates for mutations with F(t) > 19.1. The green line and points represent a typical outcome according to the Poisson distribution for 2048 individuals. In blue is the observed incidence. The grey line displays the absolute difference between modeled and observed.

Given the unexpectedly high rate of multiple polymorphisms in single individuals, we next sought to validate predicted sequence alterations as bona fide polymorphisms. To establish an F(t) threshold that would allow discrimination between true sequence alterations and noise in our screening protocol, a set of 69 predicted mutations with F(t) scores between 1.1 and 146.0 was chosen for Sanger sequencing analysis. Sixteen of these mutations were validated (Fig. 1, Table 2). No false positives were observed among predicted mutations with an F(t) score higher than 19.1, while only 10 out of the 31 predicted mutations with an F(t) score between 7.7 and 19 were validated (Fig. 4a). Comparing the typical Poisson distribution to the observed mutations with an F(t) score higher than 19.1 (Fig. 4b, Supplementary Table S1), the number of individuals with multiple mutations more closely fits the expected Poisson outcome indicating an increasing reliability of mutation discovery as the F(t) score increases.

**Table 2:** Summary of mutations validated through Sanger sequencing and their predicted effects on protein function.

Gene	Position in TILLING fragment	F(t) score	Zygosity	Effect on protein sequence	Predicted severity of mutation
HaLHY	C3741T	146.0	Homozygous	S333F	PSM
HaLUX3	A89G	87.2	Heterozygous	D15G	PSM
HaELF4_2	G391A	38.1	Heterozygous	D122N	NSM
HaFT4	C792T	30.3	Heterozygous	P76S (conserved domain)	PSM
HaELF3_3	C1187T	24.2	Heterozygous	S73F (conserved domain)	PSM
HaPIN3/4/7_1	C956T	19.1	Homozygous	P305S	PSM
HaLUX2	C227T	17.8	Heterozygous	Q68* (conserved domain)	PSM
HaGA2OX	T1816C	17.0	Heterozygous	S188P (conserved domain)	PSM
HaPIN3/4/7_4	A3786G	16.1	Heterozygous	Y588C (conserved domain)	PSM
HaLUX3	C389T	14.9	Heterozygous	S115F	PSM
HaLUX3	A530G	14.6	Heterozygous	E162G	PSM
HaPIN3/4/7_4	G1347A	14.3	Heterozygous	G281D	PSM
HaLUX2	C813T	11.8	Homozygous	P263L	PSM
HaPIN3/4/7_3	G2147T	10.7	Heterozygous	G581C (conserved domain)	PSM
HaLUX2	G542A	8.5	Heterozygous	A173T (conserved domain)	NSM
HaPIN3/4/7_2	C1467T	7.7	Heterozygous	R214W	PSM

All 32 predicted mutations with an F(t) score less than 7.7 proved to be false positives. Thus, all the validated mutations had an F(t) score equal to 7.7 or higher, making this a conservative threshold for selection of mutations for further analysis.

Among the 16 confirmed mutations, most are GC to AT transitions (69%), followed by AT to GC transitions (25%) and GC to TA transversions (6%). Fifteen of the 16 validated mutations are predicted to cause changes in the amino acid sequence of the protein, while the last is predicted to cause protein truncation. Among the missense mutations, 13 are predicted to be PSM and 2 NSM. 44% of these mutations fall in regions corresponding to a protein domain predicted to be highly conserved by the Conserved Domain Database (Supplementary Fig. S4). Three mutations are homozygous while the remaining 13 are heterozygous (Fig. 1, Table 2). In particular, the homozygous mutations are located in the putative *HaLHY*, *HaPIN3/4/7_1*, and *HaLUX2* genes and have F(t) scores of 146.0, 19.1, and 11.8, respectively (Table 2). All of these mutations are predicted to be PSM by CAMBa2. Thus TILLING by sequencing is an appropriate method for the identification of potentially deleterious alleles in sunflower, a primarily outcrossing species.

**Figure 5:**
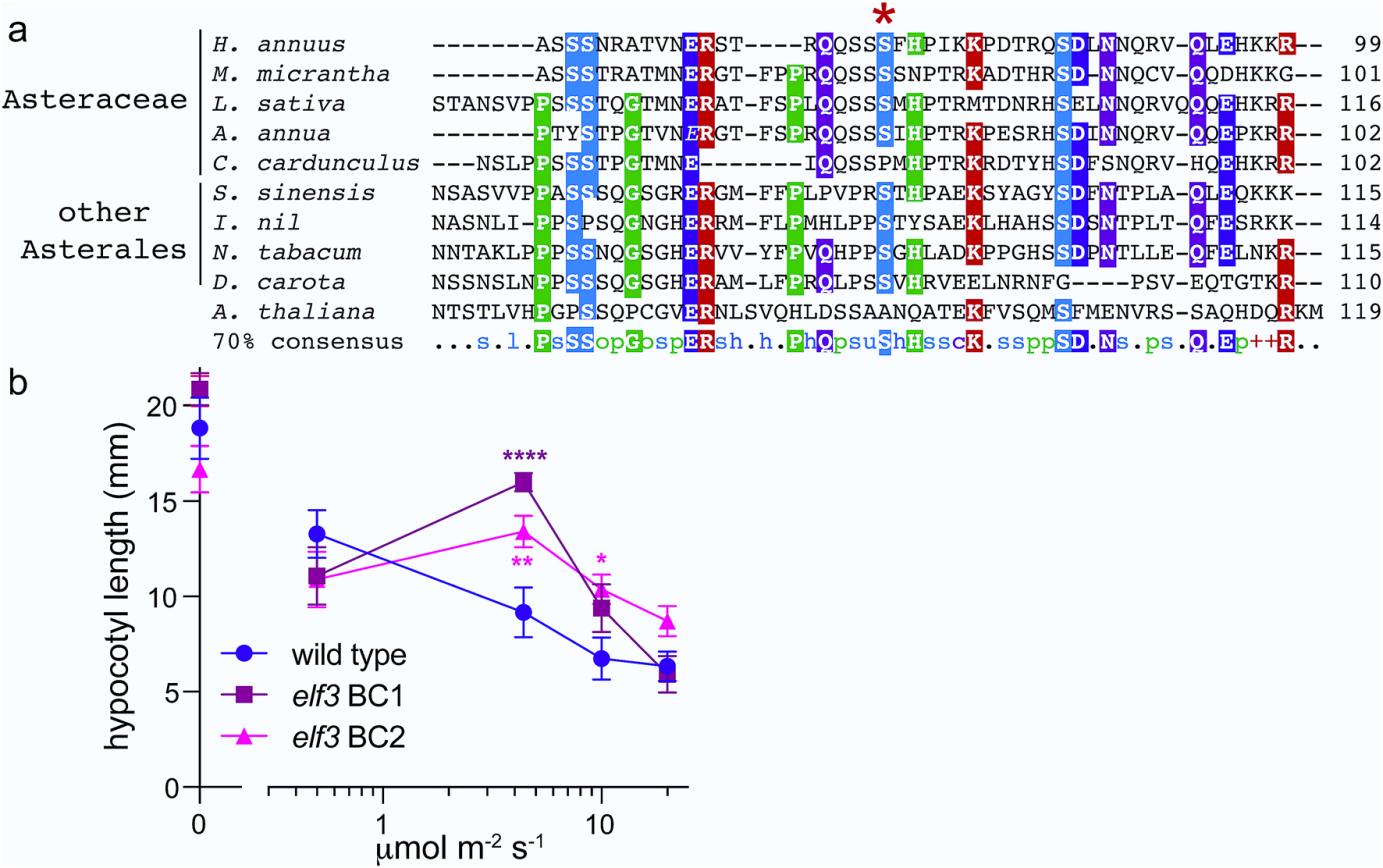
** (a)** Sequences of the predicted proteins most similar to HaELF3_3 from the indicated species. Starred residue corresponds to S73 in HaELF3_3. (**b)** Hyposensitivity to light in sunflower with an induced variant in *HaELF3_3*. Seedlings were maintained in constant darkness or monochromatic red light of the indicated fluence rates for 10 days and then hypocotyl length was measured. BC1 and BC2 represent homozygous populations of *elf3_3* S73F plants backcrossed once or twice, respectively, to the parental genotype. Mean ± SEM is plotted. Significance assessed using t-tests with FDR correction; **** < 1 e−4, ** < 1 e−2, * < 0.05.

We next wished to assess one of the mutant genotypes for a phenotype that might be associated with variation in the target locus. One of our confirmed novel mutations occurs within a conserved residue of HaELF3_3 (Table 2, Fig. 5a), a sunflower homolog of the clock- and light-signaling gene *ELF3*^43^. Both knockout mutations and natural variants of *Arabidopsis thaliana ELF3* have been reported to have defects in inhibition of hypocotyl elongation in response to red light^44,45^. We therefore examined this process in the parental GV342 genotype and two backcrossed *elf3_3* S73F lines. No significant difference in hypocotyl length was observed in etiolated seedlings or plants grown under very low light. However, at intermediate fluence rates the mutant hypocotyls are significantly longer than those of wild type (Fig. 5b), revealing decreased sensitivity of the *elf3_3* S73F genotype to light relative to control plants. These data suggest that the induced mutation, which occurs at a site conserved in many Asterales ELF3 homologs, may impair protein function.

## Discussion

The release of the *Helianthus annuus* whole genome sequence^4^ represents a significant advance for genomic and physiological studies in this species and will facilitate the identification of genes of interest involved in an array of important biological processes. However, both functional analyses of fundamental biological processes and crop improvement require the identification of variants in genes of interest. Here, we report a step towards this important goal, with the screening of a TILLING population for variants in 21 genes of interest and the identification of 16 verified mutations.

We first verified genomic and cDNA sequences for sunflower homologs of genes involved in auxin transport, flowering time regulation, circadian clock, and centromere function (Fig. 1, Supplementary Data S1). cDNA corresponding to *HaLUX*, *HaELF3* and *HaELF4* homologs was detected in samples collected in the evening but not in the morning or midday. Conversely, detection of cDNA corresponding to a putative *HaLHY* gene was detected in the samples collected in the morning and midday, but not in the evening time point. These findings support the proposed functions of these genes, since Arabidopsis *LUX*, *ELF3* and *ELF4* are evening-phased clock genes and *LHY* is a morning-phased gene^14^. Only genes with cDNAs that could be amplified through PCR were included in our analysis, since the knowledge of the exact ORF sequence is essential for the correct evaluation of mutation effect.

Although both *H. annuus* and *A. thaliana* underwent different episodes of polyploidization involving the whole genome^46,47^, multiple homologs were found corresponding to each Arabidopsis gene in sunflower. This may be due to a different extent of gene retention causing the different number of sunflower homologs. Other Arabidopsis single-copy genes were found corresponding to multiple paralogs in sunflower, such as the *FT* gene^23^. This makes mutant characterization tricky since the paralogs could have retained the same function although a possible functional differentiation is not to be excluded. Therefore, a deep analysis of all the identified genes is necessary to elucidate the roles of each paralog and the presence of possible interactions among them.

The TILLING by sequencing strategy^30^ is an effective method to detect rare mutations in large populations. We used this approach to screen about 58 kb of coding sequence in 2048 mutagenized individuals with genomic DNA pooled through a tridimensional scheme. Our data analysis revealed an overall high level of sequence variation attributable to sequencing errors that was particularly noticeable in regions with low sequencing coverage (Fig. 2a–c). The observed high variation in sequence coverage was expected, since fluctuations are common in high throughput sequencing methodologies and they could be influenced either by events during library preparation, such as a partial loss of random nicking activity of fragmentase, or during bioinformatic analysis, e.g. misalignment due to reference sequences with high similarity.

CAMBa2^40^ was used for mutation detection since it both allows the identification and evaluation of candidate mutations taking the pooling scheme into account and addresses problems associated with high-variance in sequence coverage. Alternative programs are also available for the same purpose, although they are less effective with high-variance samples^36,39^.

An important aspect of TILLING by sequencing experiments is determining an appropriate F(t) threshold that separates likely true mutants and false positives. We considered three factors when choosing candidate mutants for validation: the F(t) probability score, the type of mutation, and the position of the mutation. Putative mutations with high F(t) score, predicted deleterious effects on protein function, and localization in conserved protein domains were prioritized over the others. The high number of detected false positives identified in our follow-up experiments could be due to multiple factors. A first problem could result from the concurrent analysis of genes belonging to the same family and the presence in the population of undetected diverged alleles and duplications. Misaligned reads would negatively influence mutation detection^48^. In the future, this could be ameliorated by further optimizing alignment parameters and increasing read length. Alignment problems can be further exacerbated by sequencing errors, which are intrinsic to each sequencing platform; the sequencing error rate of the Illumina technology used in this experiment is estimated to be ≥ 0.1%^49^.

Another feature of the discovered set of mutations is the frequency of GC to AT transitions, the typical result of G-alkylation generated by EMS mutagenesis^30,50^. In rice, Arabidopsis, wheat and other crops characterized by TILLING, the frequency of these types of alterations is typically over 90%^30,31,37,40,50^. About 69% of our validated mutations are canonical GC to AT transitions. Although lower than the expected value of > 90%, this percentage is much higher than the 26% observed in all high- and medium-quality candidates. This low frequency suggests either high noise and/or contamination of the population by naturally-arising polymorphisms. Noise is expected to affect distribution among individual plants in a stochastic fashion. On the other hand, genetic contamination is expected to produce a pattern where some individuals display more mutations than expected by chance. Comparison of modeled and observed data (Fig. 3a,b, Supplementary Table S1) reveals a pattern consistent with some genetic contamination. Most TILLING populations have some contaminants, but the highly outcrossing habit of sunflower^51,52^, even if lessened by selection during breeding, may have contributed. Indeed, although sunflower heads were bagged during breeding to prevent cross-pollination, nevertheless it is possible that some cross-pollination occurred. Genetic contaminants could be also attributable to natural heterogeneity in the non-mutagenized seed stock, as shown in previous studies^35,53^. If some of the mutations correspond to natural allelic variants, variation in the primer-binding regions may help explain the relatively low confirmation rate of mutations in our follow-up Sanger sequencing experiments.

Decades of research on natural and induced genetic variation has led to the assembly of large collections of well-characterized genetic materials that are a fundamental resource for understanding plant development and physiology. Forward and reverse genetics approaches have been particularly successful in linking genes to phenotypes. The TILLING technology represents a powerful reverse genetics technique for mutant identification. The use of EMS as a mutagen is particularly advantageous as this can generate allelic series in targeted genes, potentially facilitating the study of essential genes and structure/function based analyses of novel proteins. Most TILLING populations were developed on predominantly autogamous species, such as wheat, rice, and tomato. This makes the self-fertilization of M_1_ plants to produce the M_2_ population an easy process. Our TILLING by sequencing studies in sunflower, a primarily allogamous species, have revealed an unexpectedly high incidence of multiple polymorphisms per individual, suggesting a higher degree of genetic contamination than found in previous TILLING populations. Thus our results emphasize that the generation of TILLING populations in primarily out-crossing species requires extensive precautions not needed when working with autogamous species. In addition to demonstrating how the challenges posed by outcrossers can be addressed effectively, our detection and validation of mutations that are likely to affect protein function in multiple regulatory loci highlight the importance of this sunflower population as a genetic resource for the study of genes of interest in this important crop species.

## Methods

### Plant material and TILLING population

Leaves and stems from 10 day-old seedlings of the sunflower (*H. annuus* L.) wild-type inbred line GV342 were used for DNA and RNA extraction. For RNA extraction, leaves and stems were also collected from 2 month-old plants and the samples were collected at 3 different time points: ZT0 (dawn), ZT8 (mid afternoon) and ZT16 (dusk) in long day conditions. The sunflower TILLING population developed by Sabetta et al.^28^ was used for mutant screening. The M_2_ population consisted of 3,651 plants from which the DNA of 1,152 plants had been previously extracted^28^.

### Candidate gene isolation

Candidate genes were identified using the Nov22k22 sunflower genome assembly (http://www.sunflowergenome.org/) and the NCBI database (http://www.ncbi.nlm.nih.gov/). The *Arabidopsis thaliana* protein sequences of selected genes (https://www.arabidopsis.org/) were used as queries in tblastn searches against the draft genome of sunflower. The presence of an open reading frame (ORF) inside the scaffold was predicted by FGENESH (http://www.softberry.com) and the corresponding protein sequence was obtained using the ExPASy translate tool (http://expasy.org/tools). Moreover, a phylogenetic analysis was carried out through Phylogeny.fr^54^, using PhyML 3.0 software^55^ that is based on the maximum likelihood method; ClustalW and Gblocks were respectively used for the multiple alignment and the alignment curation. Finally, in order to confirm the coding sequence (CDS) prediction, a comparison with the sunflower HaT13 and Ha412T4 transcriptomes (https://www.heliagene.org) was also done.

Genomic DNA from 10 day-old seedlings of the wild-type inbred line GV342 was extracted according to Sabetta et al.^28^ and the concentration and quality of genomic DNA were determined using SYBR Green I dye fluorescence and electrophoresis on 1% (w/v) agarose gels. Since accurate ORF sequences are crucial for the evaluation of how a mutation affects gene function, cDNAs corresponding to the identified candidate genes were sequenced as well. To this end, leaves and stems from 10 day-old seedlings and 2 month-old plants grown in long day conditions were collected at 3 different time points: ZT0 (dawn), ZT8 (mid-afternoon) and ZT16 (dusk). Total RNA was extracted following the TRIzol Reagent RNA isolation protocol (Life Technologies, USA) and the concentration and quality were determined by spectrophotometry and electrophoresis on 1% (w/v) agarose gels. cDNA was synthesized using SuperScript II Reverse Transcriptase (Life Technologies, USA) following the manufacturer's instructions. In most cases, the first-strand cDNA was obtained with an oligo(dT) primer, while, due to the low-abundance of *HaPIN3/4/7* and *HaCENH3* genes, the first strand synthesis had to be primed using gene-specific primers targeted to the 3′-UTR.

Both genomic and cDNA sequences of the candidate genes were amplified by PCR and then cloned into the pCR8/GW/TOPO TA vector (Life Technologies, USA) and transformed into One Shot Chemically Competent *E. coli* (Life Technologies, USA) cells. Ligation and transformation were both conducted following the manufacturer's instructions. The plasmids were extracted following the method described by Birnboim and Doly^56^ and DNA concentration was determined through spectrophotometric measurements and confirmed by electrophoresis on 1% (w/v) agarose gels. The cloned fragments were sequenced through Sanger sequencing. Sequences were analyzed using MultAlin^57^ and the sequence alignment editor BioEdit^58^.

To increase the size of the screening population to 2,048 M_2_ plants, an additional 896 M_2_ plants were also subjected to DNA extraction. The genomic DNA was transferred into 64 wells of 96-well plates and normalized to 5 ng/μl.

### Primer design, template pooling and PCR conditions

To amplify the genomic and cDNA sequences from wild-type plants and to screen the TILLING population, primers were designed using Primer3^59^. Forty-three genomic gene-specific primer pairs were designed according to the instructions given by UC Davis TILLING Core (http://tilling.ucdavis.edu/index.php/Primer_Design_and_Testing_Guide) to amplify the entire gene sequences. These primers were also used for the TILLING population screening. Twenty-eight cDNA gene-specific primer pairs complementary to the putative 5′ and 3′ UTRs or putative exons of the identified candidate genes were also designed. Amplification was carried out using TaKaRa Ex Taq DNA Polymerase Hot-Start Version (Clontech, USA).

Genomic PCR amplifications were carried out in 30 μl reactions containing 15 ng of DNA, 1X Ex Taq buffer, 0.2 mM dNTPs mix, 0.3 µM primers mix, 0.025 µl of 5 U/µl Taq polymerase. PCR program was established by UC Davis TILLING Core (http://tilling.ucdavis.edu/index.php/Primer_Design_and_Testing_Guide). cDNA amplification was performed with the same conditions using 50–100 ng of cDNA and 0.5 µM primers mix. The program used was the following: 95 °C for 2 min; 40 cycles of denaturation at 94 °C for 20 s, annealing at T_m_ for 1 min, extension at 72 °C for 2 min; and the final extension at 72 °C for 5 min. PCR products were visualized on 1% (w/v) agarose gels.

The pooling strategy was the same described by Tsai et al.^30^. All 2,048 genomic DNA samples were arrayed into 96 well plates using a tridimensional scheme. Samples were divided into four sets of 512 individuals each arrayed into 24 DNA pools of 64 samples each through a tridimensional model to form 8 row pools, 8 column pools, and 8 dimensional pools. In this way, each sample was located in three different pools and the identification of single mutation was reliable. Each pool was amplified with 36 genomic DNA primers against the candidate genes. 15 ng of input pooled DNA (corresponding to about 30× sampling per individual) was used as template for each PCR reaction.

Amplicons were quantified through SYBR Green I dye fluorescence, normalized to the lowest concentration and pooled into a unique PCR pool plate so that each well contained the same genomic DNA pool amplified with all the primer pairs. The pooled samples were then purified using the Agencourt AMPure XP—PCR Purification Kit (Beckman Coulter, USA) following the manufacturer's instructions with some modifications: Agencourt AMPure XP was added to the PCR reaction using a volume ratio of 1:1 instead of 1.8:1 (AMPure : PCR reaction) and after 40 μl of elution buffer was added to each sample, the plate was incubated for 5 min at room temperature instead of 2 min as suggested by the manufacturer.

### Illumina library preparation and sequencing

Samples were fragmented using NEBNext dsDNA Fragmentase (New England BioLabs, UK) and the libraries were prepared using the KAPA Hyper Prep Kit Illumina (KAPA Biosystems, USA) following the manufacturer's instructions with only one modification: 96 different eight-base barcoded adapters were used instead of the Illumina original ones, in order to pool more libraries with different barcodes into one sequencing lane. Two sequencing lanes were used for sequencing, so the 96 libraries were pooled into two groups, each of them comprised of 48 libraries. The libraries were sequenced using a HiSeq2500 sequencing machine (Illumina, USA) in rapid run mode with 100 bp paired-end reads by the Genomics Sequencing Laboratory (GSL) at the University of California, Berkeley, USA.

### Bioinformatics and mutation validation

Reads in paired-end sequenced Fastq files were divided into their original genomic libraries based on the sequenced index reads (with one mismatch allowed) using the allPrep-8.py Python script (http://comailab.genomecenter.ucdavis.edu/index.php/Barcoded_data_preparation_tools). This script also removed sequencing reads with ambiguous nucleotides (N’s), trimmed sequence reads using a sliding window (5 bp) average quality with a cutoff of Phred 20, and removed sequencing reads with a minimum length of less than 35 bp. The bwa-doall.py script, in conjunction with SAM tools^60^, was used to align the sequencing reads to the target amplicons sequences (http://comailab.genomecenter.ucdavis.edu/index.php/Bwa-doall). This script performed the alignment through the BWA software^61^. The default parameters for paired-end alignment were used. The resulting SAM file contained mapping positions for each read. A .bam file was generated from the .sam file and the .sorted.bam files were generated subsequently. Two Python scripts, mpileup.py and mpileup-parser.py (http://comailab.genomecenter.ucdavis.edu/index.php/Mpileup), were used with default parameters to produce a table of coverage by position across all libraries. Next a parsed mpileup file, a simplified version of the mpileup file in which the information is summarized and compacted, was generated. A frequency plot for each target amplicon was generated using another Python script (generate_frequency_change_graphs_for_each_gene_1.6.py).

Finally, an updated version of CAMBa^39^, CAMBa2, was used for mutation detection. CAMBa2 addresses problems associated with high-variance data sets and is not biased by sequence read depth^40^. The inputs to CAMBa2 were the parsed pileup table, the pooling scheme file, and the reference sequences (amplicon sequence, genomic sequence trimmed to begin at the translational start codon, and coding sequence) for each target sequence. CAMBa2 processed each position along a queried amplicon fragment separately, considering each possible configuration (assignment of mutations to individuals) that satisfied the assumption of at most one mutation per individual. It then computed the probability of obtaining the observed base calls under each candidate configuration, assuming a binomial model of sequencing error. CAMBa2 incorporated the prior probability of each configuration in order to compute the posterior probabilities of those configurations using Bayes’ theorem. CAMBa2 identified the mutant individual, assigned a probability score, and finally produced a list of candidate mutations with their predicted effects. JMP software (SAS, USA) was used for statistical analysis of libraries, including the coverage and the nucleotide variation assessment.

A set of mutations was selected for validation. Each mutant was amplified with primers corresponding to the region of the putative mutation. The PCR conditions were the same as described above. The amplicon was purified through the following protocol: 100 µl of absolute ethanol and 3 µl of 3 M sodium acetate pH 5.2 were added to the PCR reaction. After incubation at -20 °C overnight, the samples were centrifuged for 20 min at 13,000 *g*. Then the pellet was washed with 100 µl of 70% ethanol and the precipitate was collected by centrifugation for 5 min. The pellet was air-dried for 30 min and dissolved in 30 μl of nuclease-free water. The purified amplicons were quantified using spectrometry and sequenced by the Sanger method.

Comparison of HaELF3_3 protein sequence with that of other Asterales was performed as follows. The best hit to the HaELF3_3 protein sequence (accession number XP_022035531.1) in each of the below species was identified in the NCBI database using a blastp search. Accession numbers of HaELF3_3 homologs are: *Mikania micrantha*, KAD4384841.1; *Lactuca sativa*, PLY90075.1; *Artemisia annua*, PWA94212.1; *Cynara cardunculus* var. *scolymus*, XP_024984823.1; *Camellia sinensis*, XP_028086475.1; *Ipomoea nil*, XP_019179335.1; *Nicotiana tabacum,* XP_016513806.1; *Daucus carota* subsp. *sativus*, XP_017229176.1; and, *Arabidopsis thaliana*, CAA0371864.1. Sequences were aligned using Clustal Omega^62^.

### Plant phenotyping

*elf3_3* S73F plants were backcrossed once or twice to parental GV342 to generate BC1 and BC2 populations, respectively, and homozygous *elf3_3* S73F families were identified using a dCAPS marker. The *ELF3_3* locus was amplified by PCR (F primer = 5′ AAGTACACGTCAACAGTCCTCTT 3′, R primer = 5′ CCGGTTTTGATTTGTTCCAG 3′) and then digested using the EarI restriction enzyme. For hypocotyl assays, seeds were surface sterilized, stratified for two days, and then germinated on moist paper. After germination, seedlings were transferred to 500 cm^2^ culture plates half filled with media (½ Murashige-Skoog media, 1% phytoagar) and plates were grown vertically at a constant 25 °C in the indicated light conditions for 10 days. Monochromatic red light (658 nm peak wavelength) was provided by LEDs (XtremeLUX, Campbell, CA). Plate positions were randomized every 3 days to minimize position effects. After 10 days of growth, plants were removed from the plates, scanned, and hypocotyl lengths measured using ImageJ^63^.

## Supplementary Information

Supplementary Dataset.

Supplementary Information.
