# Supplementary material for: A TILLING by sequencing approach to identify induced mutations in sunflower genes
Source: Sci Rep. 2021 May 10;11:9885. doi: 10.1038/s41598-021-89237-w (PMC8110748; doi:10.1038/s41598-021-89237-w)
Supplement: Supplementary file 2 — Supplementary Information. [file 41598_2021_89237_MOESM2_ESM.pdf]

**A TILLING by sequencing approach to identify induced mutations in sunflower genes**

Valentina Fanelli[1,2*], Kathie J. Ngo[2], Veronica L. Thompson[2], Brennan R. Silva[2], Helen Tsai[2], Wilma Sabetta[3], Cinzia Montemurro[1], Luca Comai[2], Stacey L. Harmer[2*]

[1] Department of Soil, Plant and Food Sciences (DiSSPA), University of Bari Aldo Moro, 70124 Bari, Italy

[2] Department of Plant Biology, University of California, Davis, CA 95616, United States of America

[3] National Research Council, Institute of Bioscience and BioResources – IBBR, 70124 Bari, Italy

***** valentina.fanelli@uniba.it, slharmer@ucdavis.edu

# Supplementary Information

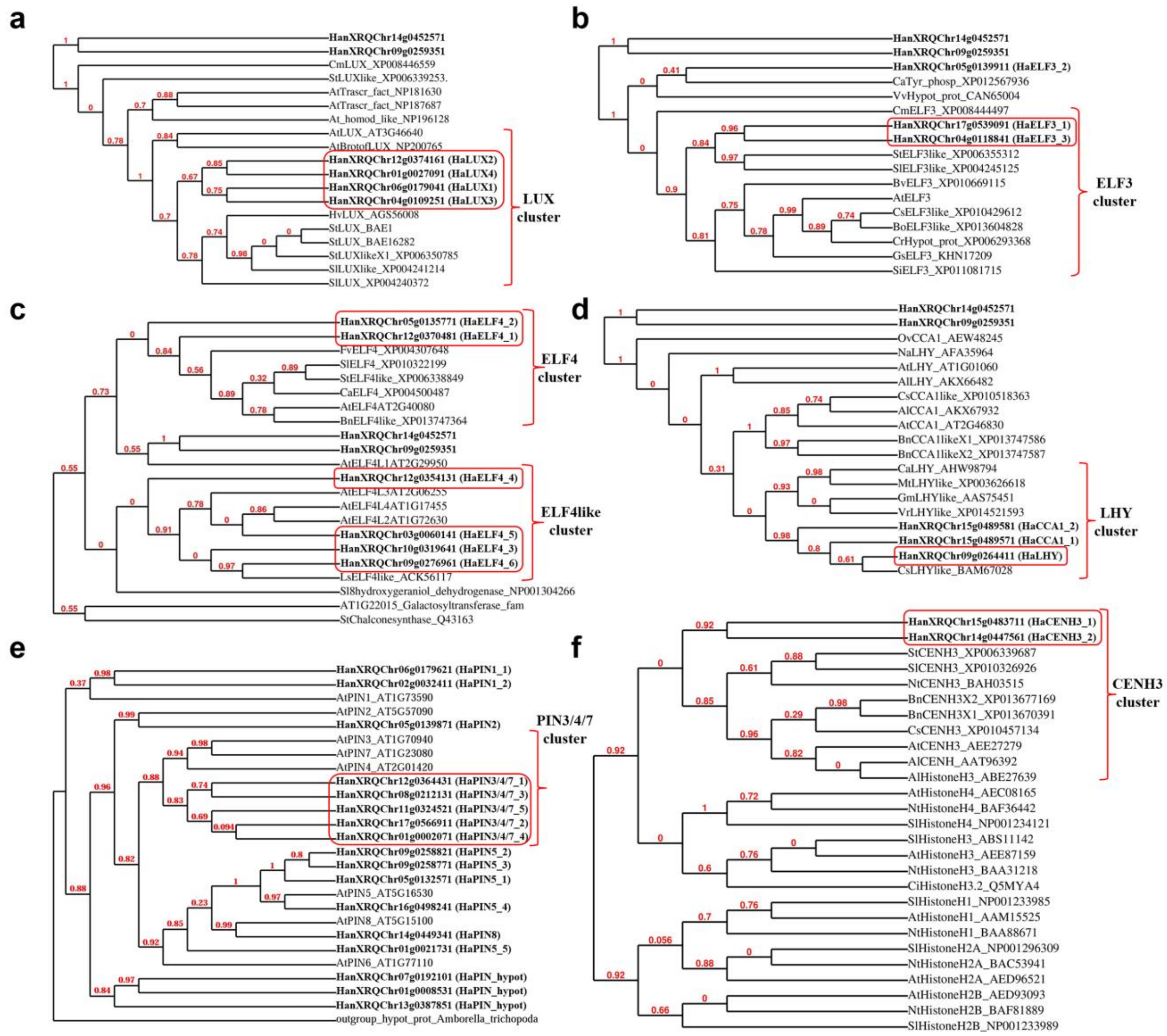


**Supplementary Figure S1.** Phylogenetic analysis performed on candidate genes *LUX* (**a**), *ELF3* (**b**), *ELF4* (**c**), *LHY* (**d**), *PIN3/4/7* (**e**), and *CENH3* (**f**). The amino acid sequences of the indicated proteins were aligned using ClustalW, curated using Gblocks, and a maximum likelihood phylogenetic tree constructed using PhyML (default substitution model; 100 bootstraps) using phylogeny.fr software (http://www.phylogeny.fr/version2_cgi/index.cgi). Bootstrap support is indicated. Prefixes indicate the following species: Han, *Helianthus annuus*; At, *Arabidopsis thaliana*; Cm, *Cucumis melo*; St, *Solanum tuberosum*; Hv, *Hordeum vulgare*; Sl, *Solanum lycopersicum*; Fv, *Fragaria vesca*; Ca, *Cicer arietinum*; Bn, *Brassica napus*; Ls, *Lactuca sativa*; Vv, *Vitis vinifera*; Bv, *Beta vulgaris*; Cs, *Camelina sativa*; Bo, *Brassica Oleracea*; Cr, *Capsella rubella*; Gs, *Glycine soja*; Si, *Sesamum indicum*; Ov, *Hordeum vulgare*; Na, *Nicotiana attenuate*; Al, *Arabidopsis lyrata*; Mt, *Medicago truncatula*; Gm, *Glycine max*; Vr, *Vigna radiate*; Nt, *Nicotiana sylvestris*; Ci, *Cichorium intybus*.

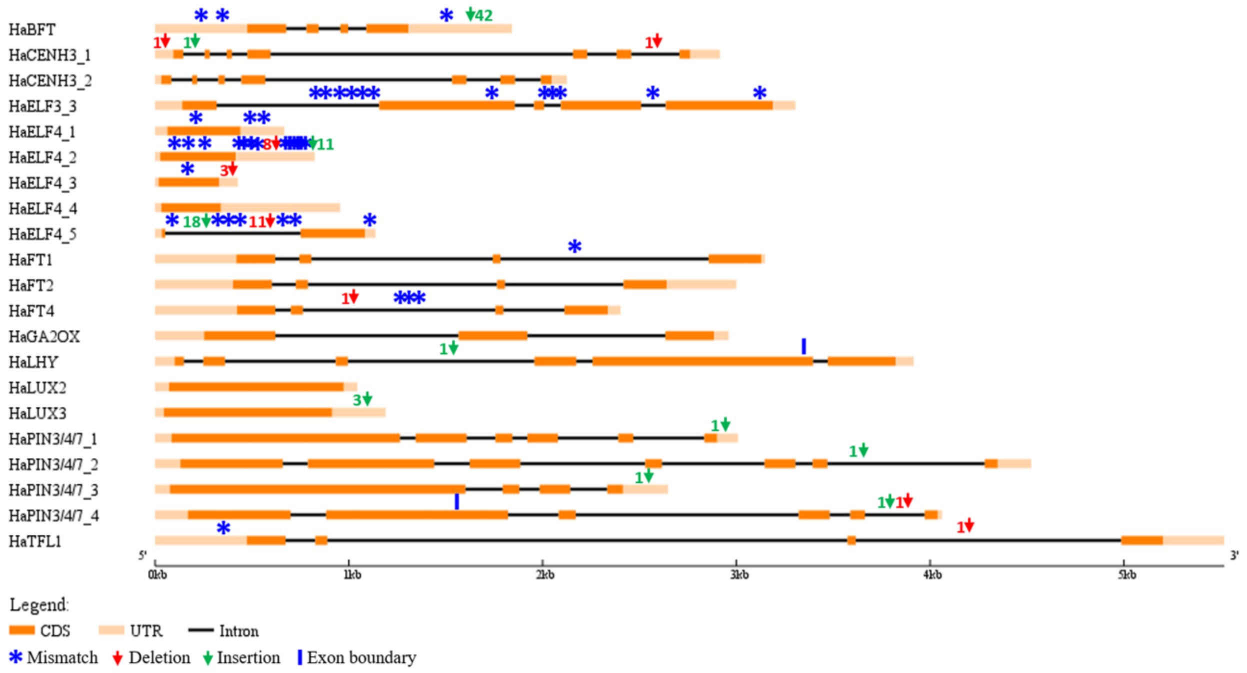


**Supplementary Figure S2.** Differences between the publicly available sequences and genomic and cDNA sequences obtained in our study. Reference consists of sequences available in NCBI database and Nov22k22 sunflower genome assembly (http://www.sunflowergenome.org/). Genomic and cDNA sequences were obtained through amplification and Sanger sequencing of candidate genes in 10-day-old seedlings of the sunflower wild-type inbred line GV342. Mismatches, deletions, insertions, and differences in exon boundaries found in our sequences compared to reference sequences are shown. The numbers of bases inserted or deleted relative to the reference sequences are indicated.

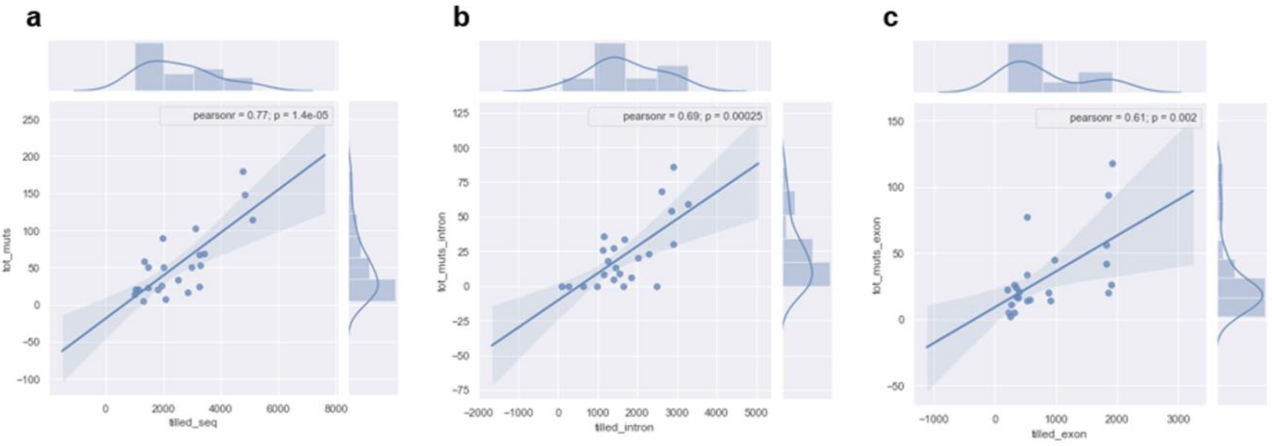


**Supplementary Figure S3.** Correlation of detected mutations to the total tilled sequence length (**a**), mutations in introns to total intronic sequence space (**b**) and detected mutations in exons to exonic sequence length (**c**).

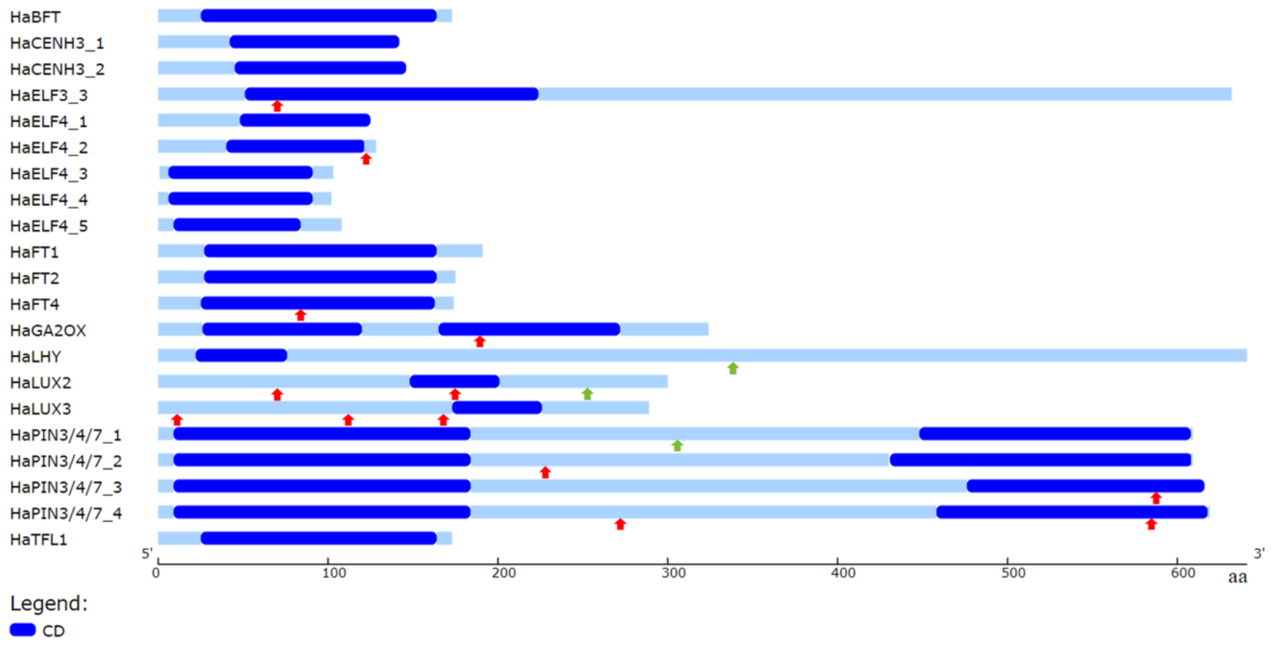


**Supplementary Figure S4.** Conserved domains identified in putative proteins and position of validated mutations. Dark blue regions indicate the detected conserved domain, while light blue regions indicate the sequence of protein in which no putative conserved domains have been detected. Red and green arrows point out the position of validated heterozygous and homozygous mutations respectively.

| Mutations per plant | Medium and high confidence, F(t) > 2 | | | High confidence, F(t) > 7.69 | | | Very high confidence, F(t) > 19.1 | | |
|---|---|---|---|---|---|---|---|---|---|
| | Observed incidence | Poisson distribution | Absolute difference | Observed incidence | Poisson distribution | Absolute difference | Observed incidence | Poisson distribution | Absolute difference |
| **0** | 1285 | 1184 | 101 | 1865 | 1847 | 18 | 2023 | 2021 | 2 |
| **1** | 526 | 649 | 123 | 165 | 191 | 26 | 23 | 27 | 4 |
| **2** | 161 | 178 | 17 | 12 | 10 | 2 | 2 | 0 | 2 |
| **3** | 48 | 33 | 15 | 5 | 0 | 5 | 0 | 0 | 0 |
| **4** | 17 | 4 | 13 | 0 | 0 | 0 | 0 | 0 | 0 |
| **5** | 8 | 0 | 8 | 0 | 0 | 0 | 0 | 0 | 0 |
| **6** | 1 | 0 | 1 | 0 | 0 | 0 | 0 | 0 | 0 |
| **7** | 1 | 0 | 1 | 0 | 0 | 0 | 0 | 0 | 0 |
| **8** | 0 | 0 | 0 | 1 | 0 | 1 | 0 | 0 | 0 |
| **9** | 0 | 0 | 0 | 0 | 0 | 0 | 0 | 0 | 0 |
| **10** | 1 | 0 | 1 | 0 | 0 | 0 | 0 | 0 | 0 |
| **less than 3** | 1972 | 2010 | 241 | 2042 | 2048 | 47 | 2048 | 2048 | 8 |
| **3 or more** | 76 | 38 | 38 | 6 | 0 | 6 | 0 | 0 | 0 |
| **p-value** | 2.29E-10 | | | 1.36E-21 | | | 0.9778 | | |

**Supplementary Table S1.** Observed and modeled distribution of mutation numbers in 2048 screened M2 individuals. P-value was calculated from Chi-square test on two bin grouping mutants with less than 3 mutations and with 3 or more mutations.

**Data S1.** Genomic and cDNA sequences obtained through Sanger sequencing.
